# The smell of polluted leisure in the Anthropocene: a qualitative arts-based approach to studying sport, eco-emotions, the senses, men, and the environment

**DOI:** 10.3389/fspor.2025.1650956

**Published:** 2025-08-12

**Authors:** Clifton Westly Evers

**Affiliations:** School of Arts and Cultures, Media Culture Heritage, Newcastle University, Newcastle upon Tyne, United Kingdom

**Keywords:** sport, environment, senses, eco-emotion, masculinity, environmental activism, arts-based method, surfing

## Abstract

This paper explores eco-emotions and sensory experiences, particularly the sense of smell, of men engaging in sports while navigating pollution along an industrial coastline. Using sensory ethnography that includes experimental arts–based perfume workshops and smell walks, I make a case for bringing analysis of men and sport, the senses, and the environment into closer dialogue with eco-emotions, arguing that doing so has the potential to widen our understanding of men's embodied sporting lives and any associated role in environmental sports activism. Eco-emotions refer to emotional responses to environmental challenges, for example eco-hope and eco-anxiety in relation to climate change and pollution. The paper provides evidence of how men in a toxic community navigate eco-anxiety, precarious hope, and eco-disgust through “polluted leisure” olfactory competencies alongside other practical skills that enable moments of pleasure while enduring a polycrisis. The working-class white men in the study demonstrate sophisticated sensory knowledge and complicated eco-emotional lives as they live with and adapt to pollution in ways that trouble any simple binary of activism and ignorance or apathy. The men perform intentional and unintentional mundane, daily, idiosyncratic, tactical, quiet, and intimate actions that add to forms of environmental sport activism that may be taken and paid attention to.

## 1 Introduction

They come to the cold North Sea. Caramel-hued foam marbles the wave faces, clinging to their neoprene skins. The foam tastes of chemical alchemy, not salt. The surfers dive, rise again. Seagulls call. So do horns from the port. The air they breathe tells stories of industrial profit, failure, and heritage. The smell of the sea ebbs beneath the sharper acrid accord of the petrochemical cracker plant's emissions wrinkling noses and making them wince. Their bodies soak in both pleasure and toxicity.

This essay uses a polluted leisure framework to explore eco-emotional and sensory experiences of sport in the Anthropocene. Climate change and pollution are altering environmental conditions such as temperature and air and water quality, frequency of extreme weather event and natural disasters, and fundamentally changing how sporting and leisure activities are embodied, done, imagined, organised, sensed, and felt (1, 2). While sports and leisure researchers examine affects and emotions (3, 4) and how emotions and the senses shape human–nature sporting relationships (5), there' is still work to be done in understanding how eco-emotions are playing out across sporting bodies and in relation to the senses. In this paper, I make a case for bringing analysis of sport, the senses, and the environment into closer dialogue with eco-emotions, and which can add to our understanding of environmental sport activism. Eco-emotions—including, for example, eco-grief, eco-anger, eco-anxiety, eco-hope, and solastalgia (place-related sadness, mourning, grief)—are ecologically situated and attuned emotions. The case is made through a focused study of men's embodied sporting lives on a polluted industrial coast.

This paper proceeds through five main sections. Following this introduction, I link studies of sporting emotions with eco-emotions, clarifying my understanding of emotions (including affect) and eco-emotions, with a key takeaway message being that eco-emotions operate both individually and collectively. I then situate the study regarding other work of sport (understood as polluted leisure) and the senses. Next, I explain the methodology, detailing the sensory ethnography approach and the perfume workshop and smell walks used to explore sensory and eco-emotional experiences of men and sport. The analysis section is an ethnographic storytelling that explores the relationships between pollution, eco-emotions, and the senses (focusing on smell) for surfers in a toxic nature and toxic community. The conclusion synthesises these insights and makes some recommendations.

What is polluted leisure? Polluted leisure is “the embodied, sensorial, emotional, intellectual, spatial, and technological busyness of pollution—material and social; harmful and non-harmful; actual and perceived—occurring through leisure” (6). Pollution simultaneously creates risks for sport and leisure activities, while also enabling new forms of sport and leisure experiences and enjoyment. For instance, waste dumped on the seabed can form new surf breaks, while polluted urban areas provide unique architectural features and spaces where skateboarders innovate culture, find belonging, and play. The paper adds to a body of work with an interest in the sensory life of polluted leisure and sport (7). In this paper, I focus on smell. This sense carries considerable emotional weight because olfactory awareness involves not only the immediate, sometimes involuntary or unwelcome detection of odours, but also complex cognitive and embodied memories that they trigger (8, 9). Smells directly shape people's everyday feelings of the environment and vice versa (8).

Smell plays a key role for embodied sporting lives (10, 11). Research about sport and the senses favours sight, taste, smell, touch, and hearing. During the ethnography, I noted how vision, touch, taste, and hearing may not detect some pollution that is discernible by the nose. I am not seeking to advocate for sensory bias. Embodied sporting lives and their eco-emotional and environmental entanglements can only be fully understood within a multi-sensory context. Rather, I use smell to provide analytical focus to examine how sporting bodies, senses, and pollution interact to formulate eco-emotional experiences in a “toxic nature” and “toxic community.” When combined with other studies, this research will contribute to understanding eco-emotions, sport, and the environment through a multi-sensory lens.

By toxic nature I mean an environment where pollution has become an intrinsic part of the ecosystem, fundamentally shaping its visual character, functioning, and the physical, emotional, and cognitive experiences of all inhabitants, both human and non-human (12). By toxic communities, I am referring to those sited next to polluting industries, which tend to be poorer and sometimes racialised (13). Some people are more available to be poisoned (14), while privileged groups get to live in denial (15). The paper adds to our understanding of how sport and leisure involve sensorially adapting to, living with, and emotionally experiencing waste, pollution, and climate change (1, 2, 6, 16–19).

The empirical fieldwork informing this paper's arguments focuses on surfing along an industrial coastline in England. The participants have many stories and sensual, deeply embodied knowledge about pollution. Surfing is what has been called “lifestyle sport” (variously termed also as action sports, extreme sports, nature sports, or adventure sports).^1^ Examples of such sport and leisure include surfing, outdoor swimming, skateboarding, parkour, BMX, climbing, and snowboarding. Researchers have identified unique environmental embodied and sensory sporting lives in these sporting communities (6, 7, 17, 20–25). Surfing has also been called an archetypal “nature sport,” which refers to those sports that centres on environmental elements such as geological formations, wave energy, and atmospheric forces like wind (26). The experience of surfing as nature sport is a complex sensory process of soaking in material, elemental, social, and cultural relationships (26, 27). It is relatable to the Japanese Shinrin-yoku (“forest bathing”), which appreciates the taking in the forest through the senses (28). Nature sports happen as part of “sensescapes,” with smell playing a key role in such (8, 29). Nature sport sensescapes foster ecological sensitivities and pro-environmental behaviours through sensory immersion (30–32). To date, research about surfers and environmental activism has focused on conservation, surf break management, and organisational/institutional activism of surf-based environmental NGOs (25, 33, 34). This paper contributes not only to sport and leisure studies broadly, but also to the lifestyle and nature sport subfields.

Historical and persistent narratives portray surfers as environmentally conscious with strong environmental activist traditions and stewardship capacities (25, 33–36). However, despite the nature sensescapes, many surfers lack ecological awareness or concern, paradoxically damaging nature, while seeking to connect with nature (37). For example, building resource-intensive artificial wave park facilities, manufacturing and consuming petrochemical-derived equipment (e.g., surfboards and wetsuits), and participating in energy-intensive tourism practices that perpetuate colonialism (6, 25, 33–35, 37–42). Clifton Evers (6, 16, 43–45) argues that surfing is polluted leisure. This paper contributes to a critical surf studies subfield (46).

## 2 Analytical framework

### 2.1 Sport, leisure, environment, and pollution

The increasing influence of climate change and pollution on sport and leisure life has seen scholars turn their attention to law, governance, environmental activism, climate justice, biodiversity, and policies of sustainability (e.g., infrastructure, waste, clothing, carbon emissions, travel) as well as politics (2, 20, 47–50). Institutionalised sport sustainability efforts may, though, create new injustices and vulnerabilities. For example, environmental management proposals for surf-break protection can exclude and marginalise Indigenous peoples and seascape knowledge (51–53). Sports management researchers are pulling together a subdiscipline they call “sport ecology” to galvanise attention to and action to address both the environmental impact of organised sport and the environment's impact on organised sport (19, 54).

I have found myself interested in environmental sporting experiences that do not make headlines or even “count”—moments outside organised sport, sport management, industry contexts, or elite athletic circles. Like anthropologist Elizabeth Povinelli, I am interested “in forms of suffering and dying, enduring and expiring, that are ordinary, chronic, and cruddy rather than catastrophic, crisis-laden, and sublime … the quasi-events” (55). This study centres the experiential, mundane, and daily expertise of sporting bodies in toxic natures and communities. These are bodies that have a troubling kinship with pollution, undergoing a polluted leisure enskilment that involves attuning to pollution, an intimate living *with* it to do sport and leisure. As Carter et al., explain, embodied sport is a “sensory ecology” that creates specialist bodily knowledge (56). A polluted leisure enskilment is a multi-sensory, more-than-human, and eco-emotional embodied process of familiarising with pollution through repeated exposure and adaptation to what will now do what it wants with us (44, 57).

Polluted leisure is premised on a more-than-human approach to research that involves a recognition that non-humans—such as pollution—co-shape sport and leisure bodies, sensescapes, emotional life, institutions, politics and policies, values, practices, and activism. As environmental humanities scholars Olga Cielemęcka and Cecilia Åsberg put it: “we are in nature, and nature, polluted as it may be, is in us” (58). Polluted leisure brings into relief relationality across time and scale, and interconnectedness and shared agency with the non-human (59). Other sport and leisure scholars are using similar theories, such as New Materialism, to argue the same (20). I am particularly interested in how the ubiquitous presence and shared agency with pollution, which is indifferent to human concerns, forces us to make meaning and imagine sport and leisure with it not only cognitively and physically but sensually and emotionally.

Pollution and climate catastrophes are catalysing new sporting assemblages^2^ of sport and leisure (1, 6, 17). These assemblages are creating specialised sporting skills, new sporting technologies, new risks but also pleasures, alternative forms of environmental expertise, strange tactics of endurance through urban play by marginalised groups, reimagined sporting identities, reappropriation and repair of discarded sporting equipment and contaminated spaces, challenges to spatial governance and access, as well as new forms of place attachment, creative expression, and spiritual connection (1, 6, 17, 20, 22, 43–45, 55, 57, 62, 63). As Glenney observes in the skateboarding context, polluted leisure is productive of ways of being “suitable for our Anthropocene age” (57). Understanding the sensory and eco-emotional dimensions of those sporting assemblages still requires work.

### 2.2 From sporting emotions to eco-emotions

Men's emotional embodied sporting lives shape and are shaped by institutions, cultures, socio-economics, environment, media, and events (60, 64–68). Emotional sporting experiences operate simultaneously as individual and collective phenomena. They 're entangled with cultural, material, social, spatial, and political contexts. Sport carries feeling rules (69). These are socially embedded emotional norms that individuals perform, either consciously or unconsciously, to align with cultural expectations (69). Consider “acceptable” and even “expected” gendered outbursts of anger by men following sporting defeats, or the acceptance of crying in competitive sport settings when broader cultural norms might demand stoicism. The emotional lives of sport are filtered through gendered personal interpretative and ideological frameworks that change over time (70).

In this paper, when I refer to “emotion” I am using an umbrella term for an affect-emotion system. Affect refers to the embodied sensory intensities that amplify our experience of the world, manifesting as emotional states (the culturally interpreted, narrative expressions of affects) such as anger, shame, and joy that colour and deepen our engagement with each other, objects, and our surroundings (71). As Tomkins explains, “Without affect amplification nothing else matters, and with its amplification anything can matter” (72). Affect and emotion are not of different or discrete orders, they function together to influence meaning-making (73). When bodies experience the same affect and/or emotion, their relationship to affect-emotion will vary because of cultural politics (74). For example, the trope of the “angry black woman” or the acceptance of anger among working-class men as valued if “appropriately directed” are a product of racialised and gendered discourses. Also, it is worth noting that affect and emotion are not solely individual phenomena (75). They can generate solidarity and collective action, for example an atmosphere of joy and care when swimming together (62, 76). This means that people may commit to a decision or attachments because doing so is a way to *feel*, for example, like they can endure the violence of pollution or are up for doing environmental sport activism (or not). Decisions and attachments extend beyond rationality (77).

Sporting bodies in toxic communities are closer, more exposed, and vulnerable to pollution (13, 78). When it comes to having to navigate sport and pollution, some sporting bodies get to maintain or even enhance their sporting pleasures at the expense of those living in toxic communities. An example is the use of neoprene wetsuits by surfers, where chloroprene—the main chemical ingredient—has highly toxic production processes that are argued to cause cancer in communities (e.g., St John's Parish in “cancer valley,” Louisiana, USA) who must live next door to the manufacturing facilities (79). People in these communities are on the frontline of sporting eco-emotions. Denialism is not an option (15).

Eco-emotions refer to emotional responses to an environmental polycrisis such as climate change and pollution, for example, ecoanxiety, eco-anger, eco-grief, eco-disgust, eco-grief, and eco-hope (80, 81). Like other affects-emotions, people may experience more than one eco-emotion at the same time, and they can be experienced individually but also collectively. Eco-emotions are formed by broader, structural issues that cause climate change and pollution and in turn shape those structures. For example, sustainability sport initiatives or political efforts to block such eco-emotions affect people's behaviours and decision-making about the environment, others, and themselves, for example, becoming climate denialists to “ease” eco-guilt and eco-shame, withdrawing from sports participation because of eco-anxiety (80, 81). Eco-emotions can have negative impacts on mental health, physical health, and social relationships, undermining the benefits of sport and being in nature (80, 81). However, they can also enhance such benefits, as people are motivated to become activists and navigate them in creative ways so that they can continue to find wellbeing and pleasure through sport (80, 81). Positive eco-emotions, such as joy, increase the likelihood of engaging in pro-environmental sporting behaviours, while negative eco-emotions, such as anger or fear, may lead to avoidance or disengagement from sporting environmental issues (80, 81). Sporting eco-emotions are experienced differently and play out unevenly. They are entangled with interlocking, for example, colonial, racial, gendered, spatial, economic and generational privileges and oppressions (81).

### 2.3 Sport and the senses

Sporting eco-emotions are tangled up with the sensuous—taste, sound, smell, sight, and touch (82). The senses are more than physiological but socio-cultural skills used to interpret and evaluate the world (83). However, while sporting senses can be experienced individually, they are also shared. Sporting sensorium can be understood as more than human, for example, when fishing the sensescape is co-constituted by fish, water, wind, river flow, human, and more (84). Lupton et al. have shown how fitness enthusiasts who use wearable fitness trackers that provide training metrics including movement pace, distance travelled, average heart rate, and calories burned undertake a practice they call “data sensing” (85). That is, there is an assembling of self-tracking data with sporting sensory ecology. While training, fitness enthusiasts notice through a combined sensoria of feeling strain and data injury risk (86). Data sensing is a good example of how sporting senses are not simply located in the human body, but how there is what Deborah Lupton and Sarah Maslen call a “more-than-human sporting sensorium” (87).

Sporting sensorium, understanding, value, practices, and matter occur through continuous folding and unfolding of bodies, where those bodies are always moving, multiple, and more than human (65, 88). Sensory sporting ecologies shape our sporting relationships with the non-human and each other. There is no one way of sensing, only *ways* of sensing (11). Pollution forces itself into our lungs and up our noses, affecting our sporting eco-emotional state. However, pollution is not only environmental. Sport enthusiasts learn to recognise and action socio-cultural-economic divisions within sports through the culturally shaped polluted sporting senses (89, 90). The labelling of something or someone as “smelly” has historically served to stigmatise places and populations as “undesirable,” “peripheral,” “backward,” and “broken,” while simultaneously valorising others as “welcome,” “desirable,” “clean,” and “productive.” We all live different sensory lives, with some being more privileged than others (27). Consider, for example, the gendered social norm that requires women to mask their sweat during sport; the masculine validation found in the pungent aroma of sweat that creates homosocial camaraderie among men in locker rooms or fear for women; the smell of freshly cut grass that evokes classed memories of access (or lack thereof) to manicured sporting fields. Then there is how different bodies have different sense-abilities of the environment—for example, because of sight loss or autism (91, 92). Again, there is no one way of sensing, only *ways* of sensing (11).

## 3 Materials and methods

This empirical evidence for this paper is sourced from an ethnography (2015–present). The project was approved by the University Ethics committee (Ref: 13290/2020), contingent upon principles of informed consent, confidentiality, and anonymity. Consent from participants was documented either in written form or verbally recorded. Participation was entirely voluntary, and participants had the option to withdraw at any time. Throughout the study, participants have been repeatedly asked about consent and reminded of the university ethical codes of practice, a practice known as “continuous consent” (93). Participant details are available in Table 1.

**Table 1:** Participant details.

Pseudonym	Age	All-white British. Employment status:
Tim	42	Taxi driver
Phil	46	Chemical plant worker
Ron	53	Unemployed
Les	30	Unemployed
Matt	40	Unemployed
Terry	22	Shop assistant
James	35	Amazon driver
Richard	50	Council worker
Thomas	20	Bricklayer
Brett	23	Cleaner

The North East England research site—including Northumberland, Tyne and Wear, Durham, and Yorkshire coastlines—has a 200-year heavy industry history, including shipbuilding, petrochemical, manufacturing, mining, and nuclear energy (94). The UK has identified the region as a hub for a move to clean energy and industries (95). Companies in the region are now manufacturing wind turbines. Most of the communities in this region do not get any immediate benefit from this Net Zero UK industrial policy and manufacturing. There have been few new jobs to date, and most of the clean energy power generated goes to industry rather than households. The region has one of the highest concentrations of white British residents (93%) (96). Extensive deindustrialisation has undermined the historically preferred industrial, breadwinner masculine identity in the region (97–100). Embodied favoured masculine tropes of stoicism, strength, and “graft” (hard, physical work) persist (97–99). Deindustrialisation and the associated ecologically damaged context—including legacy and ongoing pollution—contribute to marginalisation and diminished health and wellbeing. In the three years from 2021 to 2023, the male suicide rate was higher in the North East and the North West regions than other parts of England (101). Locally, some men in the region seek wellbeing through sports, such as surfing, in toxic nature (102).

Surfing takes place in the North Sea, located between Europe and the United Kingdom. Since capitalism became the dominant economic system in the 18th century, industries have dredged, extracted resources from, depleted, and discharged waste into the North Sea in pursuit of surplus value. This “blue space”—aquatic environments like rivers, lakes, and coasts—could equally be conceptualised as what Paul O'Connor et al., call “grey space” due to its industrial and constructed nature, representing an ambiguous and liminal environment that provides symbolic richness for analysing the complex and often contradictory material-social-emotional-sensory dynamics of polluted sport and leisure (17, 103).

Recruitment was via a snowball model beginning with approaching local surfing community members. I am a man and have 40+ years of surfing experience, as well as being from a white, working-class background. Given my insider knowledge, I have convenient access to participants.

My focus on men for the study is because in England, men's bodies, masculinity, emotions, and sport have long shaped each other and become hegemonic arrangements (104). Another consideration is working towards addressing how men like myself are over-represented when it comes to carbon emissions, ecological damage, climate denialism, technological and institutionalised solutionism, and deep fossil fuel attachments (100, 105–108). While that may be the case, my approach to working with men to explore sport, eco-emotions, the senses, and the environment is empathetic. I am sensitive to how the participants' experiences reflect more than domination patterns and how they too must make meaning from the middle of what we 've been flung into, in all its mess (109).

When referring to masculinities in this paper, I do so through a framework that understands them as discursively produced within and across societies in ways that produce multiple mutating embodied sensuous masculinities (60, 61, 64, 65). There is scarce research about place-specific gendered perceptions of, adaptations, and responses to pollution through the lens of men, masculinities, and sport (6, 43–45). Gender-responsive policymaking and action plans must include a consideration of men masculinities if we are to move towards what Hultman and Pulé call “ecological masculinities” (100). The concept refers to practices of care-full practices and values for the environment.

For the ethnography, I conduct walking “go-alongs” following participant choice of routes, activities, and sites while doing informal interviews. Participants are given cameras, drawing pads, and audio recorders to multiply ways of expressing themselves and multi-sensorially participating in the research. I also use “wet ethnography,” involving shared in-water experiences to enable co-noticing of embodied, eco-emotional, environmental, and sensual sporting life *with* pollution (76, 110–112). I record this aquatic fieldwork using a waterproof camera and attached audio recorder. With participants, I combine what is gathered to create sensory ethnographic stories (113–115). The ethos I aim for through such a methodology done *with* the men is what feminist cultural studies scholar Elspeth Probyn proposes as a speaking

out with our selves and against our selves to catch the movement of being gendered, folding the lines between our selves so as to open up new perspectives, inscribing and bending images of our being into positions of becoming, all these are ways for me of extending the “grain” of the self: the grain of a political project of care and of hope (116).

I use reflexive thematic analysis (RTA) to analyse data, a recursive and inductive process that acknowledges the ongoing mutual shaping of data and findings between research questions, field, materials, theory, participant, and researcher aimed at generating the iterative, analytical, sensory, and interpretive storytelling (117). During analysis, I consult popular media sources and scholarly studies for cross-referencing.

Scholars interested in exploring the embodiment of sport and the senses have noted the methodological challenges for doing so (115). A popular way to explore the senses and sport is through interviews and sensory ethnographies—with autoethnography being particularly evocative and productive—of sensory knowledge about running, surfing, cycling, skateboarding, and swimming (7, 62, 65, 88, 118–121). To such, I add a perfume ABR and smell walks as new way of studying sport, eco-emotions, senses, environment, and pollution.

Arts-based research (ABR) methods multiply pathways for co-creating data and telling stories differently (122, 123). They are an approach to research that can, as Natalie Loveless puts it, *create* new possibilities and ways of living, while revealing unjust realities of the current order of things (124). ABR is the use of, for example, theatre, poetry, dance, painting, photography, film, sound recording, textile, collage, and zine-making for research purposes. Such methods have proven effective for studying and analysing with participants’ climate justice, toxic geographies, environmental challenges and activism, relationships with non-humans, and to the structures (both overt and hidden) that might control people's lives, while *creating* new possibilities and ways of living (43, 124–127). Data and analysis are treated as performative and co-created, rather than existing somewhere “out there” to be found and later analysed in isolation from context. ABR expands how sensory sport ethnographies are done. During ABR workshops, I observe and ask participants about what they are making, and participants also observe, interpret, and ask other participants what they are making. They become co-researchers, where their agency is recognised in the very grain of the methodological ethos and project. ABR has proven popular among participants who, as one explained to me, find it easier to “help others understand if I show them.” Eco-emotions and the senses are often difficult to put into words. They 're experienced viscerally: “in your bones,” “in your hands,” “in your guts,” “across your skin,” and “*up your nose*.”

As part of the ethnography, I facilitated two perfume workshops (ten participants). Participants chose—out of a selection of ABR offered—the perfume workshop approach during a period of heavy rain when agricultural runoff stench was particularly pungent on the coast, keeping people out of the water and resulting in more-than-usual e-coli-led infections and illnesses. The perfume workshops were combined with prior smell walks—go-alongs with participants to explore “smellscapes” (8, 128, 129). The smell walks involved walking or surfing through polluted locations with participants, during which we noted smells and any participant who noticed something would contextualise and explain it. These sessions lasted 1–2 h, and we conducted 20 of them, with some participants choosing to repeat the process. After each smell walk, we consulted perfume oils that I carried in my car, testing for similarities that could contribute to a final perfume representing the experiences and environment. I began carrying the perfume oils after conducting some pilot smell walks, which gave me an indication of what selection to have available. The collection was extensive—over 50 perfume oils—to avoid narrowing the choices. This was entirely self-funded. We sought not only olfactory similarity but also how certain smells might trigger emotions related to the place and sport. Examples of perfume oils I carried included birch tar (smoky, oily), bergamot (spicy), calone (fresh, aquatic), musk (warm, earthy, sweet), and cyclamen (light, floral, with slight pepper notes). The aim was to arrive at a final signature perfume that could articulate eco-emotions, polluted leisure, and sport activism. Zardini explains, there are “specific scents from activities, energy sources, aromas, spices, plants, flowers, animals and garbage, forming invisible yet present and olfactory landscapes” (130). Recalling how we interpret smells depends on relational cultural, emotional, and material conditioning and context, the smell walks and perfume workshops became opportunities to extend the grain of our selves through this sense.

## 4 Results and analysis

James and Richard slip-slide with me down the eroded muddy banks onto a muddy-pebbly beach. Richard sits on a shopping trolley that is washed ashore, his surfboard on his lap. They are showing me a favourite surfing place. It is littered with industrial waste, such as rubber hoses and plastic sheeting. Down the beach, a pipe spews red water onto the pebbles, its bloody rivulet weaving towards the sea. The pipe is fed by three ponds filled with reeds. The reeds are used by a mine remediation authority as patient workers to filter the mine water that still rises from abandoned coal shafts below. The mine water carries cadmium, manganese, arsenic, lead, and copper. All five of these metals are a public health concern. Red mud escapes the reeds and pools at the edge of the sea. Too much water, too much legacy for the reed beds to swallow. The sea must pick up the slack.

The smell of my sweat as I sit in my wetsuit mingles with the musky smell of the heavy metal sludge, with the mouldy smell of seaweed, and a burnt tar scent coming from a biomass plant less than 50 yards away behind a barbed fence. I ask James and Richard about the smell. James settles into the space like he is settling into familiar waters: “I don't mind, I'm used to it.” He gestures towards a housing estate in the distance. It' is where he lives. Richard says, “It's a bit smellier than it used to be. They've [the biomass plant] been digging up some of the beach and taking it away. I'm not sure why.”

We paddle out into the surf. Richard says “The smell's not as bad out here. It's windier.” Between waves, Richard gets nostalgic: “We used to camp overnight here and then surf first thing in the morning. It smells too much to do that now.” He catches a wave. Nostalgia is cut off.

Post-surf, as we walk back to our cars, I ask how this place makes them feel. They both say they feel calm after surfing here. Blue spaces have been shown to help people feel calm even though they may be facing difficulties in other part of their lives, mitigating economic, personal, and social challenges (131). They find a sense of wellbeing with a polluted blue space, not despite it (43). I nearly leave it there. James speaks. There is concern in his voice: “The smell could drive me away in the end.” It makes him “stress out and overthink things.” The sensescape is making James sometimes worry. I ask: “but you still feel calm here?” Richard responds instead of James: “yeah but it smells more dead now because of what they're doing.” I ask who is the “they” he is referring to. Richard answers: “The plant… it's just making you anxious. The place isn't what it used to be … you don't see the seals here anymore, there's no trees just the gorse now.” Gorse (Ulex europaeus)—a hardy evergreen shrub with yellow flowers—is an expert at not only surviving but thriving on this industrial coast. Other flora does not fare so well. James adds, “We don't even get jobs.”

The mention of anxiety was an a-ha moment. Eco-anxiety can be broadly defined as difficult feelings of fear, worry, a sense of helplessness, uncertainty, unpredictability, and uncontrollability regarding our environmental relationships, particularly concerning changes like ecological crises and pollution (80, 81). It can leap across to create an eco-anxious atmosphere that groups must navigate together as they witness and live through environmental and interconnected change, e.g., socio-economic. It does so for James and Richard. This dual framing is consistent with Lauren Berlant's observation that affect and emotion are simultaneously individual and collective phenomena (77).

Richard's mention of the seals reflects sensitivity to a sport ecology shared with non-humans. The Kunming-Montreal Global Biodiversity Framework has established that climate change and biodiversity crises are intertwined with eco-emotions such as eco-anxiety (132, 133). James's “over-thinking” indicates ontological insecurity, a deep feeling of uncertainty and uncontrollability that fundamental aspects of his existence and identity are threatened (132, 133). The surf spot sits on the precipice of becoming a site of solastalgia—a place-based ecological sensibility combining anxiety, grief, and mourning rather than joy (134). The animals and surf spot that once provided security and community connection, calm, and pleasure are now in conversation with eco-anxiety. Both James and Richard demonstrate how the senses, pollution, and eco-emotions fold into each other.

Back at the car we met two other participants—Tim and Ron—who had gone on a smell walk. I had samples of fragrance and essential oils in the trunk of the car, as well as cardboard tester strips, for them to use to explore what they all have sensed during today's outing. They smelled a few and expressed interest in scents that were “dirty,” “sweaty,” “chemically,” and “mouldy.” A floral scent smelling of coconut was added to the selection because it was the smell of Gorse flowers when crushed between fingers. They also added selections that smelled fresh and oceanic. As Tim said, “for the sea.”

A week later we gather at a local community centre for the first perfume workshop. Fragrances oils and essential oils were arranged on two tables. Essential oils are natural extracts derived from plants, while fragrance oils are synthetically created in labs to mimic natural scents or create unique blends. Both can be mixed with perfumer alcohol to create a signature scent. I explained to the group about notes. Notes are categories describing a fragrance's scent profile, typically divided into top or head (first impression; evaporate quickly), middle notes form the heart of a fragrance, and base notes provide a lingering aroma. Example notes are aquatic (top note), spicy (middle), and woody (base). The notes all work together to make up an accord (sometimes, more generally, called a chord). The men used notebooks to record their selections and document what they want to share, e.g., stories, memories, feelings.

On the tables are also objects the men brought along: a dead crab in a jar, driftwood, a rotten polystyrene box, some marram grass that grows on the dunes, a sprig of gorse, a broken piece of PVC pipe with tar residue inside it, some mud in a can, a chunk of algae, seaweed, a tangled cluster of washed-up fishing net, a used crab pot, and some plastic bags. There is meaningful sport equipment: a relatively new wetsuit boot, the scent of stale saltwater mixing with new neoprene's chemical smell; a block of petroleum-based surf wax with its manufactured sweet, “bubble gum” aroma; resin (used to make surfboards) that gives off a strong, head-hurting odour; a wetsuit bucket that one participant—Thomas—described as smelling like “bin juice” because his wet wetsuit stews in it for days.

The participants take drops from different essential oils and fragrance oils to mix and add them to vials, creating rudimentary test accords. Initially, things do not go well. I am not a perfumer. The scents become muddled and, while powerful, smell as Thomas put it, “like nothing much at all.” As the men help each other and talk, we learn more about what they are trying to achieve, and importantly, the stories the scents are intended to tell. We manage to create less muddled scents at the second workshop. We are a touch more experienced.

Terry aimed to use rotten egg (sulfuric) and tar notes to help create an aromatic scent referencing the chemical plant chimneys featured in his collages (another ABR method we use), and how a spot he surfs nearby can smell when the wind blows lightly from the west. As we stood waving different testing strips under our noses, Thomas expressed a wish that any final signature perfume must conjure up petrochemical traces in the wetsuit, and more abstractly about the petrochemical manufacturing of wetsuits. Thomas recently saw a documentary about how wetsuit manufacturing hurts communities that live next door to neoprene production facilities in Louisiana, USA. He unwittingly evokes Rob Nixon's argument in his book *Slow Violence and the Environmentalism of the Poor* that argues for revealing the occluded relationships between large economic actors and the violence they do against particular places and people “over there” (135). According to Nixon, slow violence is “a violence that occurs gradually and out of sight, a violence of delayed destruction that is dispersed across time and space, an attritional violence that is typically not viewed as violence at all” (136).

The violence of pollution and industrial activity is, however, not out of sight, out of mind for the men (78). Phil agreed with Thomas, insisting that the signature fragrance should somehow recreate the “gritty” atmosphere of industrial coast surfing. As he put it, “I want people to notice how different it is to the usual stories of surfing.” During the second workshop, Phil revealed a personal sensory and emotional puzzle:

I sort of like the smell of industry here. I do feel a bit guilty for liking it. It means my job … like, I care about the environment … but working where we make chemicals for plastic products [ethylene and LDPE, which form the building blocks of many plastics].

Phil explained how when he sees Surfers Against Sewage (SAS) stalls in a nearby town while smelling like plastic, an odour he described as somewhere between sweet and musky, he gets “embarrassed and anxious in case anyone he knows calls him out.” He agrees with what his friends in SAS are trying to achieve, but says “I can’t stop [working] so the environmental stuff does my head in.”

Phil is sensible to be cautious about his breadwinner role. The North East region has one of the highest unemployment rates in the country (137). Deindustrialisation and government austerity policies are taking away social and health services, reducing life expectancy and creating an identity crisis for men in the region (138). Benefits from Net Zero industrial policy, such as employment in clean energy industries, have only been for a few. Phil's eco-anxiety represents just one dimension of broader worries and problems people like him face. Sporting lives on this industrial coast play out in relation to a polycrisis that connects pollution, climate change, and biodiversity loss with economic hardship, political instability, health decline, and social upheaval (139). At the second workshop, Phil mentioned recently joining a working group at the plastic plant tasked with reducing emissions. He had also needed some new wetsuit gloves and bought some made of natural rubber. These actions, he said, made him feel “a little better about myself.”

Phil's statement “I sort of like the smell, but I do feel a bit guilty for liking it” reveals a cultural dissonance—a culturally informed environmental attitude-action gap—wrapped up in eco-anxiety. Phil's retreat from Surfers Against Sewage also shows a disconnect from organised environmental campaigns as they may introduce eco-anxiety that “does his head in.” The joining of an emissions reduction working group at his workplace and the natural rubber wetsuit glove purchase may seem mundane and insignificant, but they can be argued to represent how eco-anxiety can exist alongside small instances of precarious hope, which is “both a product of precarity and itself precarious, opening up new possibilities for collectively imagining and pursuing viable and meaningful futures in uncertain times” (140). We may feel different and contradictory eco-emotions at the same time (141).

Phil's smellscape and fragrance creation reflects the broader complexity of sport ecology in toxic communities—where economic survival, pleasure, environmental awareness, and politics interact in ways that resist any simple narratives. In Phil's situation, smell works as a sensory co-noticing of industrial-material-ecological-sport-work-activism relations. The meeting between smell, sport, and pollution evidence political struggle, both personal and collective. The co-noticing through smell at the workshop reminded me of Probyn's encouragement to explore such a “folding of the lines between our selves so as to open up new perspectives, inscribing and bending images of our being into positions of becoming … extending the ‘grain’ of the self” to identify and perhaps hold space to seed care and hope (116). This is an empathetic reading of Phil's circumstances.

Over at another table, Matt and Tim debate how to get their local knowledge of pollution into a fragrance. It is a tall order given the depth of that knowledge. They have a lifelong and ongoing polluted leisure enskilment. They know when to surf and when not to surf based not only on the colour of the water, the clanging of the dredge stirring up the historically contaminated riverbed, but through smell. They describe the “stench” of sewage overflows, the “bitterness” of industrial waste, and pungent “rotten egg” and “fishy” smells of agricultural runoff that affect local surf breaks. Through their polluted leisure enskilment, they develop a unique olfactory sporting competency. The contaminated olfactory competency informs their polluted leisure safety maps (44). The competency is sensitive to how smell varies with weather patterns, industrial schedules, tidal cycles, and seasonal changes.

During October and November, it rains a lot and privately owned sewage systems regularly overflow. In 2024, water companies in England spilled sewage into rivers and seas for a record 3.61 million hours (142). During the rainy season, the men rely more on the Safer Seas App (water quality) run by the Surfers Against Sewage organisation than at other times. However, they blend App data with their senses, an example of data sensing (85). According to Matt, “[name of surf spot] stinks too bad so it's best to avoid it even if the App says it's okay.” Les explained that “it's flooding more because of climate change … getting worse.” The UK Government flood and coastal erosion risk in England report agrees (143).

Matt explains that “you can sort of avoid the sewage,” but when it comes to the smell from the chemical plant “you don’t have a choice.” The olfactory reveals the vulnerability of sporting bodies, the impossibility of completely protecting oneself from pollution. This vulnerability may be productive in generating an ecological sensibility, an awareness of our responsibility for, and interconnectedness with each other and the non-human (23, 62). It may not, too. Smell speaks to the ways in which bodies—human and non-human—exceed their physical limits, binding us together as more-than-human sporting ecologies. Smell is a reminder that bodies and sport ecology are liminal, not controlled by us, and perhaps as sports researcher Andrew C Sparkes puts it, not “ours” at all (10).

The enthusiasm for trying to share their olfactory sporting competency draws attention to a resignation to a situation and a making do. The competencies as they pertain to sport and pollution represent a more complex process than simple desensitisation and passivity. The men signal towards, as argued elsewhere, a “resigned activism” (6, 12). According to Anthropologist Anna Lora-Wainright, this concept refers to a

spectrum of perceptions and practices comprising acts that may not fit the conventional label of collective environmental contention … but it also includes less confrontational and more individualised or family orientated strategies at minimizing pollution in one's immediate surroundings (12).

Surfing examples are washing with clean water post-surf, not swallowing the water, wearing ear plugs (6, 102). And now I learn, another is a polluted leisure olfactory competency. The men's olfactory competency enables the men to endure and continue to seek pleasure in toxic nature. The sensory competency not only signals a resigned activism, but how environmental sport activism can take the form of quiet, intimate, and unintentional resistance to environmental violence and injustices. The men find pleasure with pollution without allowing it to be entirely debilitating, without abandoning hard-won place-based attachments (43).

There is a long well-documented history of sport activism to identify and tackle the power relations that produce injustices (135, 144, 145). Sport activism can also manifest as a conservative force, representing efforts to preserve and protect the arrangements that produce the unity, form, and cohesiveness of normative sporting bodies, values, industry, institutions, cultures, spaces, and practices perceived to be under threat (135). Environmental sport activism encompasses a wide range of activities, for example, petition campaigns, educational initiatives, community forums, consumer boycotts, and various forms of protest such as event disruptions, strategic blockades, media-oriented performances, public rallies, and organised demonstrations. Much environment and sport research to date are on conventional, dramatic, and notable examples of activism (e.g., protest), as well as organisational efforts at achieving sustainability and stewardship.

The men do not see themselves as environmental activists. Tom said they do not do enough to call themselves activists. Tim put it this way, “we're too worn out to be activists.” I understand this weariness as not a closing down nor inaction. Rather, it is a kind of quiet, steady endurance. For members of these sporting communities in toxic nature and communities, the violence of pollution may be sensed and emotionally draining every day and connected to a polycrisis. They have a right to feel weary.

Forms of environmental sport activism are often hidden, quiet, informal, local, gradual, and difficult to detect (146). Feminist scholars argue for attention to the significance of the subtle and idiosyncratic moments of environmental care, witnessing, connection, and repair (146). The men's pollution-sensitive olfactory competency holds space for more recognised forms of activism to grow through the cracks in a current environmental—social order that has placed deep historical structural constraints upon them. What the men do through the polluted leisure competencies—including olfactory—fits Michel De Certeau's idea of tactics, unintentional as that may be. Michel De Certeau argues that paying attention to ordinary everyday practices can help us understand broader patterns, as interconnected practices can constitute political activism, knowingly and unknowingly (147). Strategies operate from a position of access to institutions, regulations, and discourses that rationally construct and demarcate a space of power by establishing and validating what “is orderly,” “reasonable,” and “knowable” about relationships between sport, bodies, activism, and the environment (particularly pollution, in my case). Tactics, on the other hand, can be considered as creative instances of poaching, interfering, subverting, transforming, eroding, and evading strategies. Tactics are actions by the powerless with or without conscious intent and however fleeting that contest, mitigate, cope with, and evade strategic arrangements that favour and legitimate the powerful.

After the two perfume workshops, I took the feedback, fieldwork notes from the workshops, and rudimentary fragrances and testing strips to a perfumer. Together we worked on iterations of different perfumes, recursively consulting the men about them. We changed fragrance profiles based on feedback, narrowing selections. We finalised a signature perfume for industrial coast surfers. The signature perfume featured a heavy smoky, burnt, tar-laden top note designed to create an immediate but fleeting industrial impression that might make someone initially wince and trigger disgust (birch tar oil was especially valuable for achieving this effect); a sharp, spicy middle note to emphasise themes of heightened awareness and noticing; and a fresh oceanic base note with salt, sweetness, musk, and seaweed-like moss to capture their love of the North Sea (calone—a synthetic aromatic compound—proved particularly effective). This structure ensures that the initial disgust from industrial scents gives way to the lingering oceanic attraction as the fragrance develops.

I want to pause here to reflect on the men's desire to provoke a wince (dissmell) and disgust. Dissmell represents a biological response to offensive odours that triggers characteristic facial expressions, for example, nose wrinkling, upper lip raising, and wincing. It is an instinctive aversion to potentially harmful substances (148). Tomkins classified dissmell alongside disgust as evolved mechanisms to protect us from ingesting or contacting objects that are potentially noxious to health (1,490). Beyond their evolved biological function, dissmell and disgust can work politically, signalling socio-culturally learned contempt and revulsion towards any objects, behaviours, and people—a process of boundary-making. This process of olfactory othering recalls how smell is shaped by culture and associated arrangements of privilege and oppression. Smell has long been used to other—unhygienic, diseased, strange, and weird—bodies, including sporting bodies (90). As anthropologist Mary Douglas explains, pollution is not merely environmental contamination but also a social othering process determining who deserves recognition as valuable or worthy to belong within communities—not only what but *who* is “matter out of place” (149). As a toxic community, the men know they are stigmatised by outsiders.

Sianne Ngai's work on “ugly feelings” is helpful for explaining the value of the wince (dissmell) and disgust for the men, bearing in mind their position on such reflects a white privilege given how smell can be used to racially other sporting bodies (150). Ngai points out that ugly feelings occur precisely in situations where agency is obstructed, where individuals and communities find themselves unable to effectively address the structural conditions that produce their vulnerabilities yet must nevertheless continue to navigate those conditions in their daily life (150). The men are simultaneously aware of the structures that produce pollution and their toxic community—a polycrisis—but are unable to eliminate such, forced to develop sensory practices that neither fully resist nor completely accept these conditions. Ugly feelings such as disgust seem to have evoked a reflexivity that is a transformative starting point for sensitivity to exploring shared vulnerability. Even as people wince, they may invite others to wince with them, prompting questions about who winces, why they wince, where they wince, and who does not have to wince. Disgust functions as a sporting eco-emotion and political action. Perhaps disgust even has the potential to trigger small steps towards ecological masculinities.

It could be argued that what the men do risks normalising environmental harm. I agree. Sometimes, it does. However, sometimes there is an extending of the grain and opening to possibilities, as per Probyn's ethos. The men's surfing with pollution represents neither denialism nor acceptance but rather a tactical and practical critical ambivalence (110). There is no simple binary of activism and ignorance or apathy.

The men are privileged compared with other groups who are disproportionately affected by intersectional oppressions and the polycrisis. Several of the men in my study would not say they are privileged. They have made comments during the ethnography consistent with a white, male victim discourse dominating UK cultural and political landscape. A couple of the men feel, because of the polycrisis, they are “under attack.” They are being coached by the media and some political parties to treat immigrants as taking the few resources left, getting a “free ride,” as threatening “their” women, and as matter out of place (151, 152). However, as I mentioned at the outset, mine is an empathetic approach to men and masculinities, and I do not conflate several of the men's position on these matters with the other men's inclusive standpoint. I work with them men to strive to practice the ethos that Probyn proposed.

## 5 Conclusion

Ethnography and arts-based research methods—particularly perfume workshops and smell walks—are helpful to make the case for bringing analysis of sport, the senses, and the environment into closer dialogue with eco-emotions. In this paper, the men and I have shown the entanglement of the eco-emotional (e.g., eco-anxiety, dissmell/disgust) and olfactory shape human–nature sporting relationships in the Anthropocene, a process occurring more intensely and urgently for toxic communities. The burden of adaptation falls disproportionately on sport and leisure communities that lack alternatives. The differential olfactory experiences reflect broader patterns of environmental intersectionality in sporting communities.

It has been shown that sport and leisure researchers can expand efforts to understand how sporting embodiment involving eco-emotions and new sensory competencies in the Anthropocene brought about by pollution and climate change, such as olfactory competencies, can add to what typically constitutes environmental sport activism and sustainability approaches. This involves recognizing and studying more tactical, intimate, daily, idiosyncratic, quiet, and resigned forms of environmental activism beyond conventional approaches (146). I suggest taking the hard-won polluted leisure and experiential sport knowledge of toxic communities more seriously and including such when working out what to do now. They are experts, as much as the officially recognised sport sustainability “expert elite.” There is the potential for scaling up insights from polluted leisure communities to inform environmental policy and governance. How might the embodied, sensory, eco-emotional environmental knowledge generated through polluted leisure contribute to sport and environment policymaking? Should that even be done? Might we fall into the trap of normalising harm?

Going forward, my hope is that this paper will prompt research examining eco-emotion, sport/leisure, environment, and activism across different sporting communities and types, geographic contexts, sense-abilities, and environmental challenges to see what else people do daily that we can learn from. Finally, given the hard-won success of the perfume workshops, I also would love to see further method creativity to help holistically attend to eco-emotions and the senses when it comes to the environment and sport.

## Data Availability

The raw data supporting the conclusions of this article will be made available by the authors, without undue reservation.

## Footnotes

^1^While contemporary western, capitalist-led surfing has historical roots in ancient Hawaiian culture, these cultures emerge from fundamentally different epistemological foundations. Hawaiian culture is grounded in a seascape epistemology that inherently connects all beings, with an emphasis on communing with the sea and nonhumans. In contrast, the surfers in my study begin from Western onto-epistemological frameworks and may occasionally reach similar understandings only after confronting the limitations and failures of their initial worldview. This study specifically examines a surfing culture rooted in Western onto-epistemological assumptions.

^2^The concept “assemblage” has been arrived at through the work of Deleuze and Guattari (60), it being a translation of the use in their work of the original French word agencement (which is a verb). The concept refers to localized processual intra- and inter-relations involving heterogeneous material and immaterial elements and forces that self-organize. Assemblages are not fixed and contained units of homogeneity, nor are they centralised or does any element have sufficient competence to determine consistently the trajectory or impact (61). The emphasis is on consecrence—growing together—from the in-between.
